# Supplementary material for: A cryptic cycle in haematopoietic niches promotes initiation of malaria transmission and evasion of chemotherapy
Source: Nat Commun. 2018 Apr 27;9:1689. doi: 10.1038/s41467-018-04108-9 (PMC5924373; doi:10.1038/s41467-018-04108-9)
Supplement: Supplementary file 1 — Supplementary Information [file 41467_2018_4108_MOESM1_ESM.pdf]

Supplementary Methods

Optimisation of DNA staining of infected erythroid cells.

Cells were stained with cypro-Ruby at various dilutions of stock dye at either 37 or 4°C. Cells were subjected to FACS analysis after further staining with appropriate fluorescently-tagged antibodies and the DNA staining pattern of the CD45$^{-/low}$, CD11b^{-} and Ter119^{+} populations analysed by FACS to determine the conditions under which intracellular parasites were not stained, as judged best by the peripheral blood plots. The optimal staining conditions was determined to be 1:2000 dilution staining at 4°C.

Infection of naïve animals with parasitised ER and EB

100 GFP^{+} ER and 100 GFP^{+} EB were cell sorted from the spleen of mice infected with 507 clone1. One hundred GFP^{+} Ter119^{+}CD44^{low} cells were sorted from the peripheral blood of the same animal. The GFP^{+} populations were injected into naïve BALB/c mice and parasitemia monitored

In vitro reinvasion assay

A erythrocyte invasion assay was performed as described previously[38]. Briefly, Nycodenz purified schizonts were stained with anti-Ter119 at a 6:100 concentration at room temperature for 3 mins before being washed with PBS (Gibco). Merozoites were released by rupturing schizonts by serially passing through a 5 µm (Acrodisc) and a 1.6 µm (Puradisc, Whatman) filter and adding to uninfected unstained blood (10% haematocrit). Cells were incubated at 37 °C and 1200 g for 10 mins before being added to schizont media. Samples were taken at various time points and analyzed by flow cytometry.

Gametocyte enrichment with sulphadiazine

Animals were phenylhydrazine-HCl (1.25 mg) treated prior to infection IP with PbGFP$_{CON}$/RFP$_{GAM.}$ Sulphadiazine was administered to the drinking water of the animals when blood parasitemia reached >5%. Peripheral blood was recovered by cardiac puncture under anesthetic 48hrs post drug administration.

Calculation of percent contribution of EB parasitemia to subsequent ER parasitemia/gametocytemia

Total numbers of parasites in intermediate and mature EB were divided by the total numbers of ER parasites detected 24 hours later, to account for the rate of ER to EB transition and this number was multiplied by 100.

Generation of 1137cl2

DNA constructs for used in the genetic modification of *P. berghei* were prepared using standard molecular biology techniques. The *rfp* gene was amplified using a standard PCR reaction and a proofreading DNA polymerase from plasmid pG0161 (unpublished). The pair of sense and anti-sense oligonucleotides had the restriction sites for XhoI and XmaI incorporated at the 5' end respectfully. Primers designed using CLC workbench 5.0 and ordered from Eurofins MWG operons (Ebersberg, Germany). To obtain the construct $pPbRFP_{gam}$ (pG403), the XhoI/XamI flanked *cfp* in pG306[1] was exchanged for *rfp*. Plasmid pG306 contains the *cfp* under the control of the promoter of the early gametocyte marker PBANKA_101270 with the *P45/48 3'utr* downstream. The sequence of the open reading frame in each plasmid was validated by DNA sequencing (MWG eurofins) using appropriate primers. 15-30 µg of verified plasmid DNA was linearised with SacII and DNA purified.

Transfection

Transfection of $PbGFP_{con}$ parasites with linearized constructs, positive and negative selection and cloning of the $PbGFP_{CON}/RFP_{GAM}$ were performed as previously described[37,39,40]. Briefly, 100ul nucleofector solution and 5-10ug linearised DNA were added to Nycodenz purified schizonts and electroporated using an Amaxa Nucleofector device set to program U-33. The parasites where injected I.V. into an uninfected animal. Pyrimethamine (70 µg/ ml) (Sigma) was administered in the drinking water of the animals one day pi and continued for 5 days. The construct's integration into the genome was checked by PCR construct (primer pairs 3' integration and 5' integration) and positive parasites were cloned before being negatively selected using 5-FC administered in the drinking water.

A.

CD71 plots

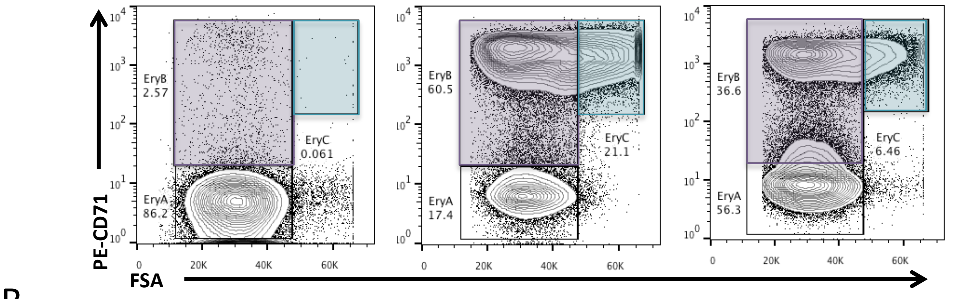

B.

CD71 MFI

	Peripheral blood		Bone marrow		Spleen	
	Ery A	Ery B	Ery B	Ery C	Ery B	Ery C
uninfected	75 ± 4	3500 ± 1618	1648 ± 79	3694 ± 494	2194 ± 255	4508 ± 614
infected	82 ± 3	1423 ± 212	1549 ± 30	4160 ± 541	1767 ± 43	2495 ± 187
	NS	NS	NS	NS	*	**

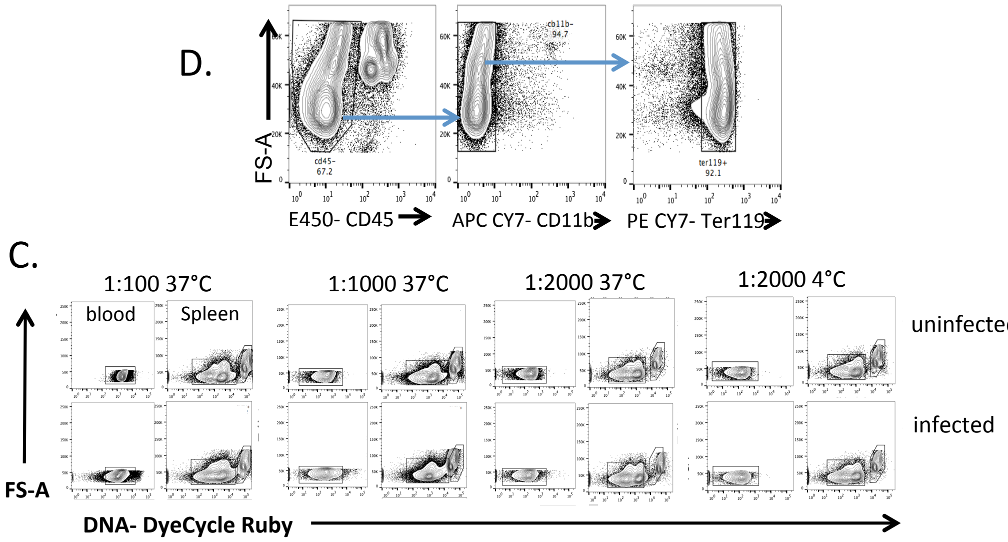

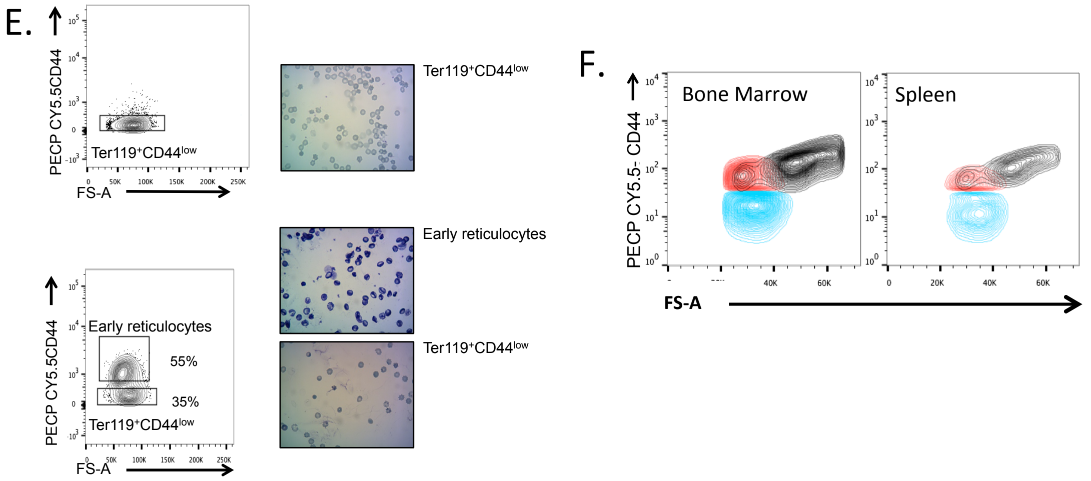

Supplementary Figure 1. Flow analysis robustly detects *P. berghei*^{GFP}-infected, Ter119^{+} CD44^{high} erythroid cells. **A**. Representative FACS plots showing CD45^{-} and

Ter119^{+} cells from tissues and peripheral blood gated against CD71 and FS-A. **B.** MFI of CD71 within uninfected and infected *P. berghei* samples. MFI values are the average intensities within the indicated gates P values are * <0.1, ** <0.01. **C.** Optimisation of DNA staining of infected EB cells. Representative FACS plots of DNA stain titration. Dye concentrations and staining temperature are indicated. For each condition there is a representative plot for uninfected (U) and infected (I) peripheral blood (Left) and spleen (Right). Cells shown on these plots are pre-gated as CD45$^{-/low}$, CD11b^{-} and Ter119^{+}. Gates were set on the uninfected samples then imposed onto the corresponding infected sample. **D.** Representative FACS gating strategy for bone marrow and splenic resident erythroid precursors in uninfected and *P. berghei* infected animals. Erythroid cells were identified by gating CD45$^{-/low}$, CD11b^{-} and Ter119^{+}. **E.** Early reticulocyte and erythroblast populations superimposed. Note the overlap in erythroblast and early reticulocyte size. **F**. Ter119^{+}CD44^{low} cells were cell sorted from bone marrow and peripheral blood. All contain pale Giesma stained cells. Early reticulocytes sorted from both organs contain dark blue cells. Representative FACS plots and imaging from uninfected bone marrow.

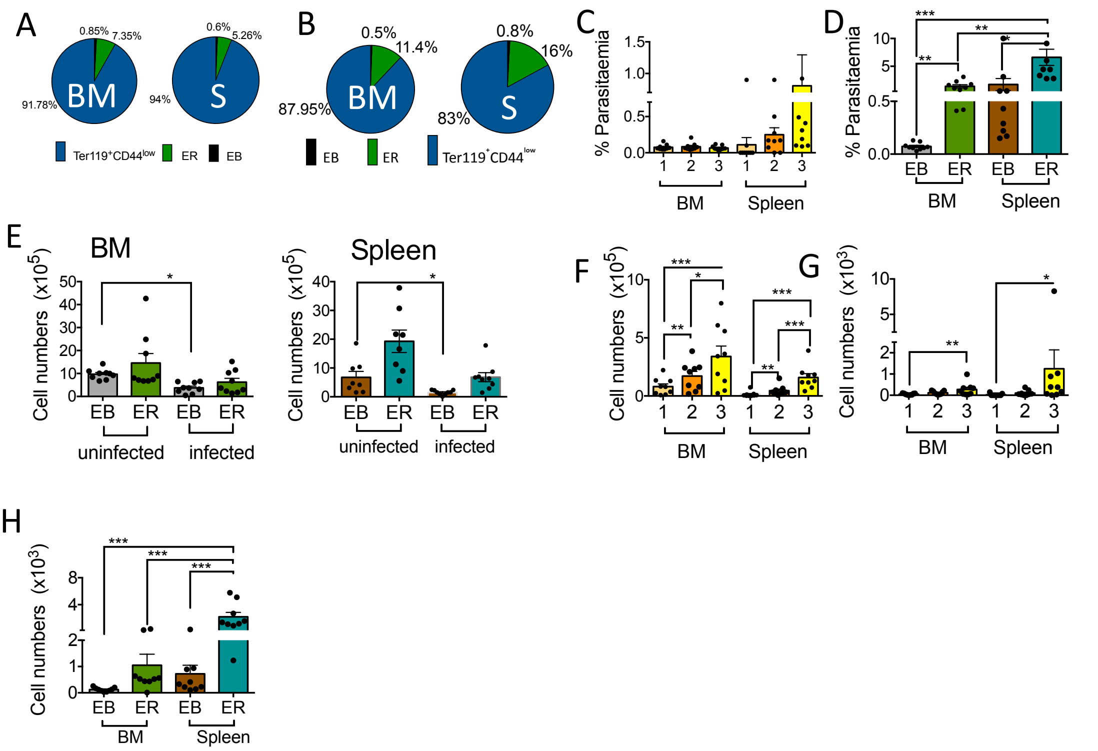

Supplementary Figure 2: Infection characteristics of *P. berghei* (PbGFP$_{con}$) in bone marrow and spleen are independent of host strain. Pie chart showing the percentage distribution of parasites within the bone marrow (BM) and the spleen (S) of BALB/c (**A**) and NIH (**B**) mice. **C**. Percentage parasitemia of erythroblast stages (1-3 as defined in Figure 1) in the bone marrow (BM) and spleen in NIH outbred mice. Error bars ± SD. n = 6. **D**. Percentage parasitemia of erythroblasts (EB) and early reticulocytes (ER) in the bone marrow (BM) and spleen in NIH mice. Error bars ± SEM. n = 6. **E**. Whole cell numbers of total erythroblasts (EB) and early reticulocytes (ER) in bone marrow (BM) and spleen of uninfected and infected NIH mice. Error bars ± SEM. n = 6. **F.** Whole cell numbers of total erythroblast stages in infected bone marrow (BM) and spleen in NIH mice. Error bars ± SEM. n = 6. **G**. Whole cell numbers of infected erythroblast stages in the bone marrow (BM) and spleen of NIH mice. Error bars ± SEM. n = 6. **H.** Whole cell numbers of infected erythroblast (EB) and early reticulocyte (ER) compartment in the bone marrow (BM) and spleen of NIH mice. Error bars ± SEM. n = 6. Significant differences mentioned above were assessed by one way ANOVA alongside Dunnett's multiple comparisons test and indicated with asterisks: * $p < 0.05$, ** $p < 0.01$, *** $p < 0.001$, **** $p < 0.0001$.

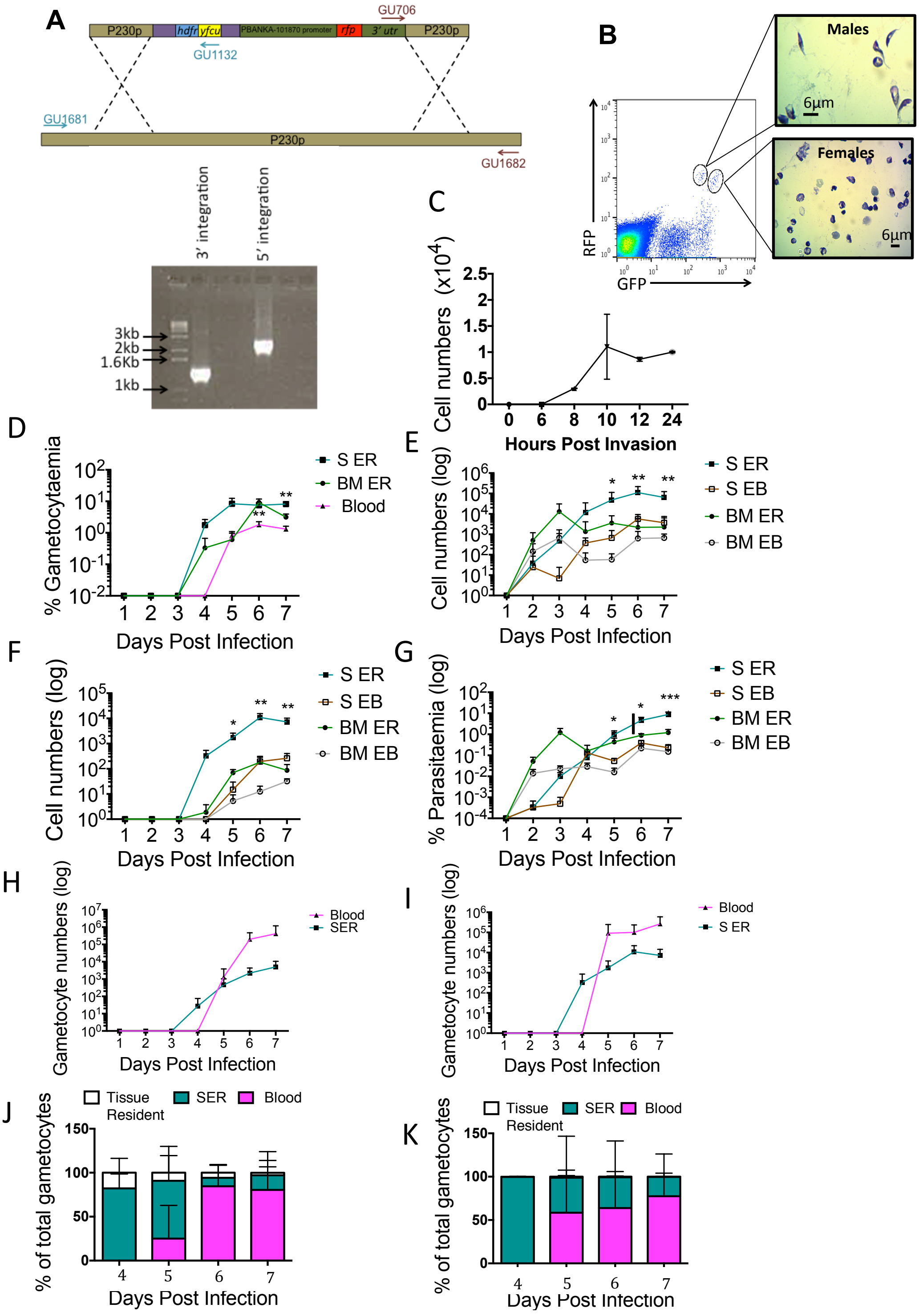

Supplementary Figure 3: Generation of gametocyte RFP proxy and gametocyte localisation within hematopoietic niches. A. Schematic of integration with genetic elements labeled. Agarose gel showing correct integration of the linearised pG403 into the *P230p* locus, creating the $PbGFP_{CON}/RFP_{Gam}$ clonal parasite line. Expected fragment sizes: 3' integration 1.3kb; 5' integration 2.5kb. **B.** Mice infected with $PbGFP_{CON}/RFP_{Gam}$ where treated with sulphadiazine and the two $GFP^{+}RFP^{+}$

populations in the peripheral blood were sorted 48hrs later. The two populations of gametocytes were activated at room temperature and the resulting gametes were visualised under a light microscope. The populations were contained exclusively male (GFP^{+}RFP^{+}) or female (GFP^{++}RFP^{+}) gametes. **C.** Total whole numbers of gametocytes present in culture post *in vitro* reinvasion assay. RFP was initially detectable by flow cytometry from 8hrs – 12hrs post invasion (n=3). **D**. Percentage gametocytemia of splenic and bone marrow derived early reticulocytes in NIH mice. **E.** Splenic early reticulocytes have a significantly higher percentage parasitemia from day 5 pi in NIH mice. **F.** Whole cell numbers of gametocytes in splenic and bone marrow derived erythroblasts of NIH mice. **G**. Whole cell numbers of asexual parasites in splenic and bone marrow derived erythroblasts of NIH mice. **H/I** Whole cell numbers of gametocytes in the peripheral blood and splenic early reticulocytes in balb/c (**H**) and NIH mice (**I**). BALB/c Day1-Day6 n=9, Day7=9; NIH all n=6. ±SEM. **J/K.** The proportion the gametocytes within the peripheral blood, splenic early reticulocytes and all remaining tissue resident compartments (BM ER, EB and S EB) contribute to the overall total gametocyte burden in balb/c (**J**) and NIH (**K**) mice. Significant differences mentioned above were assessed by one way ANOVA alongside Dunnett's multiple comparisons test and indicated with asterisks: * $p < 0.05$, ** $p < 0.01$, *** $p < 0.001$, **** $p < 0.0001$.

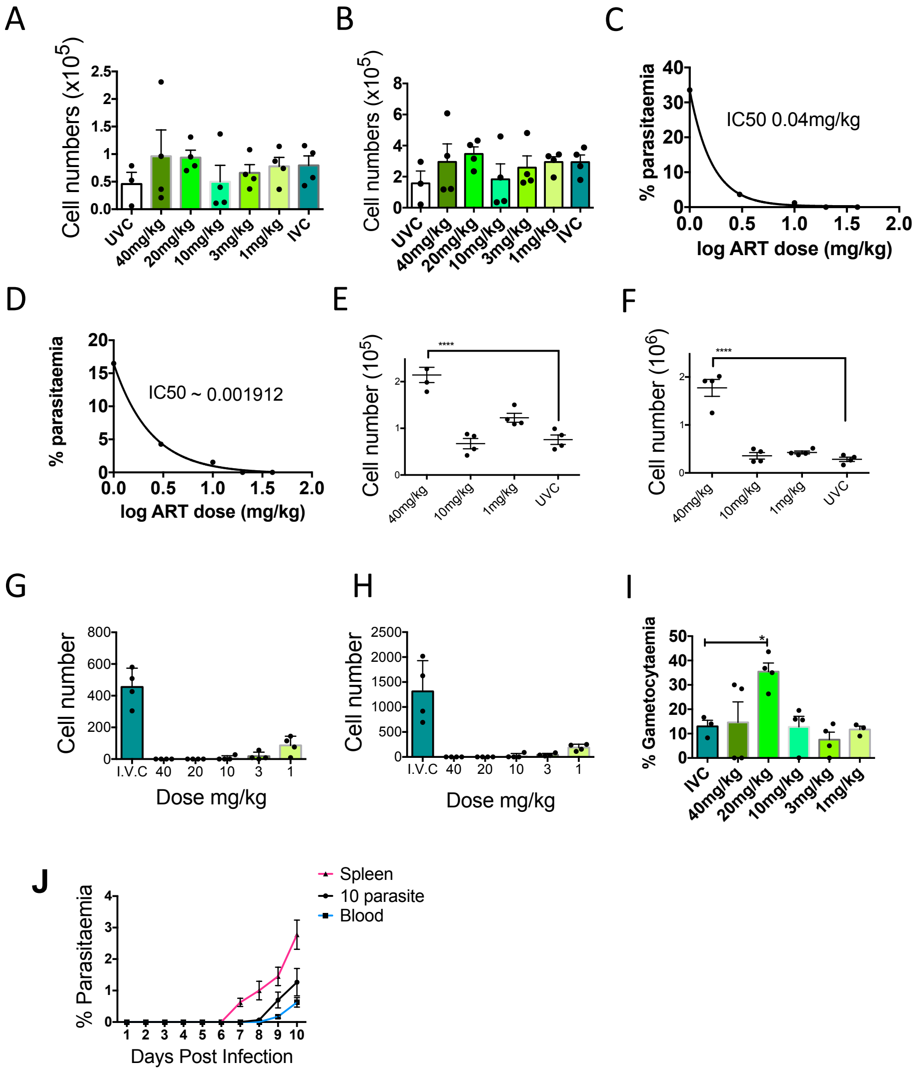

Supplementary Figure 4: Impact of drug treatment (ART and CQ) on erythropoiesis and parasitemia. ART treatment did not stimulate increased numbers of bone marrow **A.** early reticulocytes or **B.** erythroblasts compared with uninfected vehicle control (UVC) mice. Drug response curve of parasites to ART in the **C**. blood and **D.** bone marrow; graphs show percentage growth relative to infected vehicle control on day 4 post infection. n = 4. **E**. Erythroblasts and **F**. Early reticulocytes compared with

vehicle control. **G.** Parasite numbers in the erythroblasts of the bone marrow after ART treatment. **H.** Parasite numbers in the early reticulocytes of the bone marrow after ART treatment. **I**. Gametocytemia of splenic ER treated with ART. Uninfected mice treated with ART present a dose-dependent increase in splenic. **J**. The spleens were harvested from 40 mg/kg ART treated mice on day 4 pi and a single cell suspension passaged IP into naïve animals. Peripheral blood was harvested from the treated animals and also passaged IP. 10 parasites were passaged IP from infected vehicle control. n=4. Significant differences mentioned above were assessed by one way ANOVA alongside Dunnett's multiple comparisons test and indicated with asterisks: * $p < 0.05$, ** $p < 0.01$, *** $p < 0.001$, **** $p < 0.0001$.

Supplementary Table 1. MFI of CD44 within uninfected and infected *P. berghei* samples.

CD44 MFI

		Bone marrow		Spleen	
	Peripheral blood	ER	EB	ER	EB
uninfected	19 ± 14	99 ± 60	180 ± 98	45.7 ± 19	128 ± 177
infected	20 ± 14	100 ± 60	203 ± 126	45.9 ± 19	64 ± 37
	NS	NS	NS	NS	NS

Supplementary Table 2. Mean percentage contribution of EB asexual and gametocytes parasites to ER asexual and gametocytes parasite numbers in NIH mice.

Days post infection	Contribution of EB to ER asexual parasites (%) ± SD	Contribution of EB to ER gametocytes (%) ± SD
2	ND	ND
3	ND	ND
4	ND	ND
5	9.92 ± 14	ND
6	0.8 ± 0.8	0.6 ± 0.1
7	5.81 ± 5	10 ± 19

Legend: Total number of parasites in intermediate and mature EB were divided by the total number of ER parasites at the ensuring 24 hr time point then multiplying by 100. ND = Not Detected.

Supplementary Table 3. Oligonucleotides used in this study.

Pair name	Primer ID	Sequence
3' integration	GU1682	CATAAACGGTTTATTTAAAGTCATTTTTGG
	GU706	CGACTAGTCCCGGGCTTAACATTCACATATATTAATAATTTTAAT
5' integration	GU1681	TAGTTAGCTTAAATTGTCCAACTGG
	GU1132	GGTATTCTGGCAGAAG
	GU1681	TAGTTAGCTTAAATTGTCCAACTGG
	GU1682	CATAAACGGTTTATTTAAAGTCATTTTTGG
